# Feasibility of Acquisitions Using Total-Body PET/CT with an Ultra-Low ^18^F-FDG Activity

**DOI:** 10.2967/jnumed.121.262038

**Published:** 2022-06

**Authors:** Yan Hu, Guobing Liu, Haojun Yu, Ying Wang, Chenwei Li, Hui Tan, Shuguang Chen, Jianying Gu, Hongcheng Shi

**Affiliations:** 1 Department of Nuclear Medicine, Zhongshan Hospital, Fudan University, Shanghai, China;; 2 Nuclear Medicine Institute of Fudan University, Shanghai, China;; 3 Shanghai Institute of Medical Imaging, Shanghai, China;; 4 United Imaging Healthcare Co., Ltd., Shanghai, China; and; 5 Department of Plastic Surgery, Zhongshan Hospital, Fudan University, Shanghai, China

**Keywords:** ultra-low activity, image quality, total-body PET/CT

## Abstract

The present study aimed to evaluate the feasibility of ultra-low ^18^F-FDG activity in total-body PET/CT oncologic studies. **Methods:** Thirty patients with cancer were enrolled prospectively and underwent a total-body PET/CT scan 60 min after injection of an ultra-low ^18^F-FDG activity (0.37 MBq/kg). Of the 30 enrolled patients, 11 were diagnosed with colorectal cancer (CRC). PET raw data were acquired within 15 min and reconstructed using data from the first 1, 2, 4, 8, and 10 min and the entire 15 min (G1, G2, G4, G8, G10, and G15, respectively). Image quality was qualitatively assessed twice by 2 readers using a 5-point Likert scale. The Cohen κ-test was used to investigate the intra- and interreader agreement. The SUV_max_ of lesions; the SUV_max_, SUV_mean_, and SD of the livers; the tumor-to-background ratio; and the signal-to-noise ratio were measured and compared. The acquisition time for a clinically acceptable image quality using an ultra-low-activity injection was determined. In a matched-pair study, 11 patients with CRC who received a full ^18^F-FDG activity (3.7 MBq/kg) with an acquisition time of 2 min were selected retrospectively by matching sex, height, weight, body mass index, glucose level, uptake time, and pathologic types with the 11 CRC subjects in the prospective study. Qualitative and quantitative analyses were performed and compared between the 11 patients with CRC in the ultra-low-activity group and their matched full-activity controls. **Results:** Qualitative analysis of image quality showed good intra- and interreader agreements (all κ > 0.7). All the images acquired for 8 min or longer scored over 3 (indicating clinical acceptability). There was no significant difference in tumor-to-background ratio and liver signal-to-noise ratio among all the images acquired for 8 min or longer. In the matched study, no significant difference was found in the image quality score and quantitative parameters between the ultra-low-activity group with an 8-min acquisition and the full-activity group with a 2-min acquisition. **Conclusion:** An ultra-low ^18^F-FDG activity with an 8-min acquisition in a total-body PET/CT study can achieve acceptable image quality equivalent to that in the full-activity group after a 2-min acquisition.

## Visual Abstract

**Figure:**
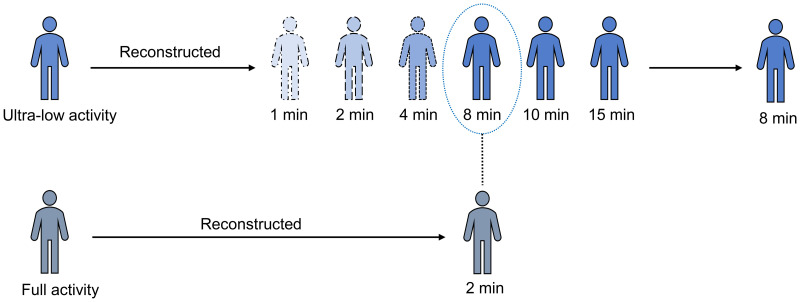

PET is an important tool for in vivo quantification of physiologic, biochemical, or pharmacologic processes. ^18^F-FDG PET is a sensitive imaging method for staging, restaging, and therapy response monitoring of malignancies (*1*–*4*). However, radiation exposure is a concern for adults, and even more so for pediatric patients, because of the summed doses from both the PET and the CT scans. According to the current procedure guideline of the European Association of Nuclear Medicine for ^18^F-FDG–based PET/CT oncologic imaging, the minimum time–mass activity product, defined as the product of injected activity and the acquisition duration per bed position, is 14 or 7 MBq·min/kg for a PET system that applies a PET bed overlap of less than or equal to 30% or more than 30%, respectively (*5*). This time–mass activity product is based on the performance of a current conventional PET scanner with an axial field of view of 15–25 cm. With the advent of the total-body PET/CT scanner with ultra-high sensitivity, the time–mass activity product of ^18^F-FDG could be significantly reduced. Previously, our group investigated the effects of a short acquisition duration on image quality and lesion detectability using the latest PET/CT scanner model, which demonstrated the feasibility of a significantly shorter acquisition time while maintaining image quality and diagnostic performance (*6**,**7*). By contrast, several studies have proposed a reduction in the injected ^18^F-FDG activity with total-body PET/CT (*8*–*10*). A recent study investigated the kinetics of ^18^F-FDG in healthy volunteers using a 10-times reduction of the injected activity (0.37 MBq/kg) in a total-body PET/CT scanner, which showed an image quality equivalent to that of full-activity imaging (*11*). However, to the best of our knowledge, no studies have investigated the effects of a 10-times reduction of the injected activity on ^18^F-FDG PET image quality in patients with various types of cancers. In the study on ^18^F-FDG kinetics, “full activity” had different definitions, 3.7 or 4.4 MBq/kg, according to the adjusted routine practice in our department. Therefore, in the present study, the lower value of 3.7 MBq/kg was used as the full activity definition. The purpose of the study was to investigate the feasibility of a 10-times reduction of the injected activity for ^18^F-FDG PET imaging in a total-body PET/CT scan for oncologic application.

## MATERIALS AND METHODS

### Patients

Thirty patients with various cancers and all referred for a total-body ^18^F-FDG PET/CT study from June to September 2020 were enrolled prospectively in the first part of this study. All patients had pathologically diagnosed malignant tumors. The exclusion criteria included lesions less than 10 mm in diameter, no uptake of ^18^F-FDG in primary lesions, disease in the liver precluding measurement of quantitative metrics in the normal liver, body mass index of at least 30 kg/m^2^, blood glucose level of more than 7.0 mmol/L, and an ^18^F-FDG uptake time of more than 70 min. On the basis of our previous work, an ultra-low ^18^F-FDG activity (0.37 MBq/kg) was administered (*11*). During the uptake phase, patients were instructed to remain quiet in a warm room for about 60 min and drink 0.5–1 L of water. In the subsequent matched study, 11 patients with colorectal cancer (CRC) who underwent full-^18^F-FDG-activity (3.7 MBq/kg) total-body PET/CT imaging were selected retrospectively from our database and matched with the same demographic and pathologic results of the 11 patients with CRC in the ultra-low-activity group; the sex, height, weight, body mass index, blood glucose level, and uptake time were well matched. The uptake procedure in the matched study was the same as that in the ultra-low-activity group. The study was approved by the Institutional Review Board of Zhongshan Hospital, Fudan University, and written informed consent was obtained from all the subjects in the prospective study. The need for written informed consent was waived for the 11 patients in the matched study given its retrospective design with anonymous retrieval of imaging data.

### Data Acquisition and Reconstruction

All patients fasted for at least 6 h before the ^18^F-FDG injection, and their level of fasting blood glucose was no more than 7.0 mmol/L. All PET/CT scans were performed in a total-body PET/CT scanner (uEXPLORER; United Imaging Healthcare) with an axial field of view of 194 cm. The PET images of the ultra-low-activity group were acquired after 15 min and then reconstructed using the first 1, 2, 4, 8, and 10 min of data by temporally down-sampling from the acquired 15-min raw data to simulate faster acquisitions, hereafter referred to as G1, G2, G4, G8, G10, and G15, respectively. The PET images of the full-activity group were reconstructed using an acquisition time of 2 min, hereafter referred to as g2. PET reconstructions were performed using the ordered-subset expectation maximization algorithm with the following parameters: time of flight and point-spread-function modeling, 3 iterations, 20 subsets, matrix of 192 × 192, slice thickness of 1.443 mm, and gaussian filter of 3 mm in full width at half maximum.

### Image Quality Assessment

The PET image quality was assessed independently by 2 readers (both nuclear medicine physicians and both with 5 y or more of experience in interpreting PET/CT images). The qualitative analysis of image quality was scored on a Likert scale of 1–5 (1, unacceptable image quality: extremely poor contrast with significant noise; 2, poor image quality: low contrast with noise; 3, acceptable image quality: moderate contrast with noise; 4, good image quality: good contrast with less noise; 5, excellent image quality: perfect contrast with minimal noise). A score of 3 indicated the minimum acceptable image quality for clinical reporting. For each patient, all the PET images were loaded into the viewer using software (uWS-MI, R001; United Imaging Healthcare). The order of the PET images was randomized by an independent operator. The readers were unaware of the patient’s demographic information, medical history, and acquisition duration. In addition, each reader reassessed the image quality 1 wk later to eliminate the memory effect, using a different order of patients and PET images. The image quality values of each reader were averaged and compared between the ultra-low- and full-activity groups.

In a separate session performed 1 wk after the second qualitative assessment, the quantitative analysis of image quality was first performed by 1 of the 2 readers by manually drawing a 2-dimensional circular region of interest (ROI) with a diameter of 2 cm on the homogeneous area of the right lobe of the liver. ROIs were placed automatically at exactly the same location and slice for the entire loaded PET series. The ROI was carefully drawn to avoid lesions and was at least 1 cm away from the edge of the liver. The SUV_max_, the SUV_mean_, and the SD were recorded. The SUV_max_ of the primary lesion was delineated at the corresponding PET transverse slice with the maximum diameter in the CT images for comparison of image datasets. The size of the ROIs was adapted to the lesion size. The liver signal-to-noise ratio (SNR) was calculated by dividing the SUV_mean_ by its SD, and the tumor-to-background ratio (TBR) was calculated by dividing the lesion SUV_max_ by the liver SUV_max_.

### Statistical Analysis

Statistical analysis was conducted using SPSS 20.0 (IBM Corp.) and Prism 6.0 (GraphPad Software Inc.). Numeric parameters are presented as the mean ± SD, and categoric variables are described as frequencies. A *P* value of less than 0.05 indicated statistical significance. The intra- and interreader agreement for the qualitative scores was analyzed using the Cohen κ-test (0.00–0.20 = low; 0.21–0.40 = medium; 0.41–0.60 = moderate; 0.61–0.80 = good; 0.81–1.00 = excellent). The Kolmogorov–Smirnov test was performed to test the normality of the objective image quality, and the Wilcoxon rank-sum test was used to compare these parameters in G1–G10 with those in G15. The Fisher exact test and an independent-sample *t* test were used to compare the categoric and numeric variables between the ultra-low- and full-activity groups, respectively.

## RESULTS

### Patient Demographics in Ultra-Low-Activity Group

The patient demographics in the ultra-low-activity group are summarized in Table 1. Thirty patients were enrolled in the prospective part of the study (20 men and 10 women; mean age of 66.10 ± 8.44 y). The average fasting blood glucose level was 5.75 ± 0.66 mmol/L, and the mean uptake time after injection was 60.97 ± 5.96 min. Diagnoses of the malignancies were confirmed using pathologic examinations.

**TABLE 1.** Demographics of Patients in Ultra-Low-Activity Group

Variable	Dataset
Sex*	
Men	20
Women	10
Age (y)^†^	66.1 ± 8.44 (48.00–77.00)
Height (cm)^†^	165.5 ± 7.25 (157.00–186.20)
Weight (kg)^†^	62.28 ± 10.2 (44.80–88.00)
BMI (kg/m^2^)^†^	22.73 ± 3.28 (15.76–29.75)
Blood glucose (mmol/L)^†^	5.75 ± 0.66 (4.80–7.00)
Uptake time (min)^†^	60.97 ± 5.96 (51.00–70.00)
Injected dose (MBq)^†^	25.53 ± 4.07 (17.76–33.67)
Primary tumor type*	
HCC and ICC	4
CRC	11
Lung cancer	1
Pancreatic cancer	3
Esophageal cancer	2
Mediastinal sarcoma	1
Bladder cancer	4
Ovarian cancer	2
Lymphoma	1
Laryngeal cancer	1

* Number of patients.

^†^Data are presented as mean ± SD followed by range in parentheses.

BMI = body mass index; HCC = hepatocellular carcinoma; ICC = intrahepatic cholangiocarcinoma.

### Image Quality in Ultra-Low-Activity Group

The subjective image quality scores of the ultra-low-activity group are summarized in Table 2. The intra- and interreader agreements were good for the subjective image quality score (all κ > 0.7). In groups with an acquisition duration of 8 min or longer, the agreement was excellent (all κ > 0.85). There was a significant difference in image quality regarding the Likert scale between G15 and the other groups (G1–G10) (*P* < 0.001). All images with an 8-min acquisition time or longer had a score over 3 and were judged acceptable for clinical reporting.

**TABLE 2.** Subjective Image Quality Score in Ultra-Low-Activity Group

Acquisition duration (min)	Image quality score			
	Likert scale score		Cohen κ-test result	
	Reader 1*	Reader 2*	Intrareader agreement	Interreader agreement
1	1.10 ± 0.31	1.14 ± 0.35	0.898 (0.694–1.000)	0.849 (0.530–1.000)
2	2.07 ± 0.37	2.10 ± 0.41	0.902 (0.711–1.000)	0.885 (0.674–1.000)
4	3.00 ± 0.38	2.90 ± 0.49	0.895 (0.688–1.000)	0.750 (0.492–1.000)
8	4.07 ± 0.53	3.97 ± 0.63	0.900 (0.701–1.000)	0.852 (0.638–1.000)
10	4.38 ± 0.49	4.38 ± 0.49	0.921 (0.738–1.000)	0.854 (0.648–1.000)
15	4.62 ± 0.49	4.59 ± 0.50	0.945 (0.812–1.000)	0.930 (0.790–1.000)

* Mean value and SD were calculated on basis of subjective scores for each patient.

Data in parentheses are 95% CIs.

**FIGURE 1.**
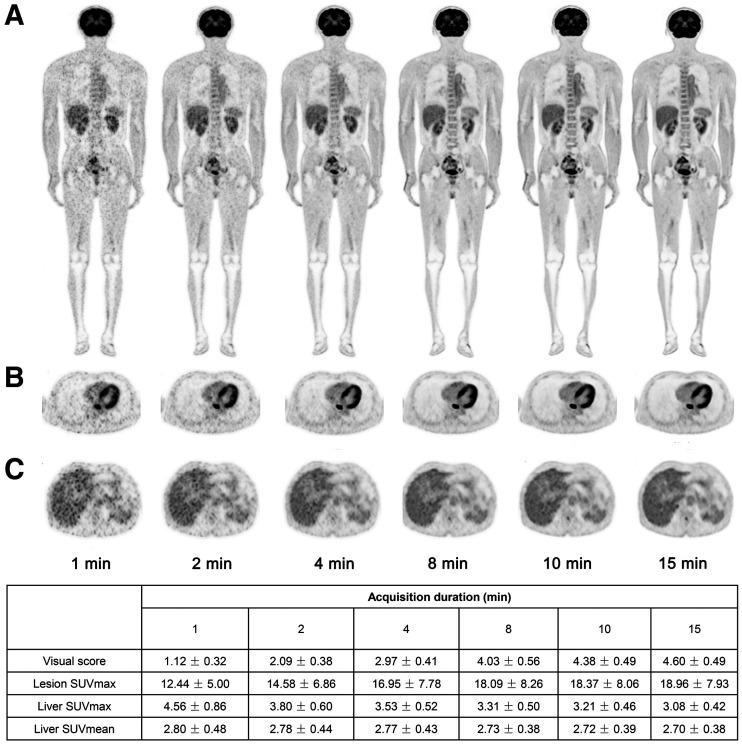
PET images of 63-y-old man with esophagus cancer. Coronal slice of whole body (A), transverse view of intense uptake of lesions in esophagus (B), and transverse view of liver (C) are shown in G1, G2, G4, G8, G10, and G15 reconstructions. More superior image quality of liver was observed in G8 than in G1 and G2 on visual assessment.

**FIGURE 2.**
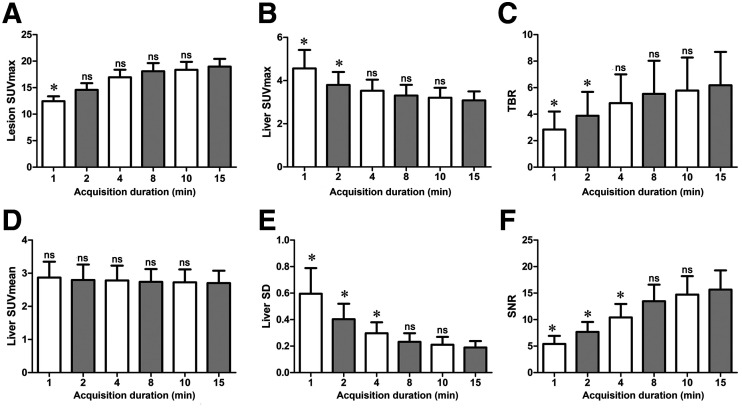
Box plot of lesion SUV_max_ (A), liver SUV_max_ (B), TBR (C), liver SUV_mean_ (D), liver SD (E), and SNR (F). Lesion SUV_max_, TBR, and SNR increased with extension of acquisition time, whereas liver SUV_max_, liver SUV_mean_, and SD decreased. Compared with G15, no significant differences for these parameters were found in G8 and G10. *P < 0.05. ns = not significant.

As shown in Table 3 and Figures 1 and 2, the lesion SUV_max_ increased with the duration of acquisition; however, the difference was significant for an acquisition time of only 1 min compared with that for G15 (all *P* > 0.05). The liver SUV_max_ decreased with longer acquisition times, and the TBR increased; however, the difference was significant for both parameters only for acquisition times shorter than 4 min (*P* < 0.05). The liver SUV_mean_, SD, and SNR are summarized in Table 4. There was no difference in the liver SUV_mean_ among all the groups (*P* > 0.05). The liver SD decreased rapidly from G1 to G15, whereas the SNR increased progressively. However, there were no statistical differences between G8, G10, and G15 (*P* > 0.05). Therefore, images from G8 had an image quality equivalent to that from G15 and were suitable for clinical reporting.

**TABLE 3.** Quantitative Image Quality in Ultra-Low-Activity Group

Acquisition duration (min)	Lesion SUV_max_	Liver SUV_max_	TBR
1	12.44 ± 5.00*	4.56 ± 0.86*	2.84 ± 1.36*
2	14.58 ± 6.86	3.80 ± 0.60*	3.89 ± 1.80*
4	16.95 ± 7.78	3.53 ± 0.52	4.83 ± 2.18
8	18.09 ± 8.26	3.31 ± 0.50	5.53 ± 2.50
10	18.37 ± 8.06	3.21 ± 0.46	5.78 ± 2.50
15	18.96 ± 7.93	3.08 ± 0.42	6.18 ± 2.52

* Significant difference compared with that in G15 (*P* < 0.05).

Data are presented as mean ± SD, based on measurement in ROIs.

**TABLE 4.** SUV_mean_, SD, and SNR of Liver

Parameter	Acquisition duration (min)					
	1	2	4	8	10	15
Liver SUV_mean_*	2.80 ± 0.48	2.78 ± 0.44	2.77 ± 0.43	2.73 ± 0.38	2.72 ± 0.39	2.70 ± 0.38
Liver SD*	0.56 ± 0.19^†^	0.38 ± 0.11^†^	0.28 ± 0.08^†^	0.21 ± 0.06	0.19 ± 0.06	0.18 ± 0.05
SNR*	5.41 ± 1.53^†^	7.67 ± 1.88^†^	10.41 ± 2.54^†^	13.46 ± 3.13	14.72 ± 3.46	15.65 ± 3.64

* Data are presented as mean ± SD, based on measurement in ROIs.

^†^Significant difference compared with that in G15 (*P* < 0.05).

### Patient Demographics of Matched Patients in Ultra-Low-Activity and Full-Activity Groups

Eleven patients (7 men and 4 women in each group) with CRC (10 well-to-moderately differentiated adenocarcinoma and 1 high-grade intraepithelial neoplasia) were enrolled in the matched study. The demographics of the patients with CRC in G8 and g2 are provided in Table 5. As expected, a significant difference in the injected dose was found between G8 and g2 (*P* < 0.001), although other variables, including sex, body mass index, blood glucose, uptake time, and pathologic classification, were well matched, without significant differences (all *P* > 0.05).

**TABLE 5.** Demographics of Patients in G8 and g2

Variable	G8	g2	*P*
Sex*			0.201
Men	7	7	
Women	4	4	
Height (cm)^†^	166.05 ± 6.48	167.82 ± 10.01	0.627
Weight (kg)^†^	62.96 ± 11.87	71.52 ± 18.22	0.208
BMI (kg/m^2^)^†^	22.75 ± 3.46	25.00 ± 3.92	0.168
Blood glucose (mmol/L)^†^	5.81 ± 0.61	5.31 ± 0.53	0.054
Uptake time (min)^†^	62.91 ± 5.50	58.00 ± 5.57	0.051
Injected dose (MBq)^†^	24.79 ± 4.44	271.21 ± 61.42	<0.001^‡^
Pathologic*			1.000
WMDA	10	10	
HGIN	1	1	

* Number of patients.

^†^Data are presented as mean ± SD, based on data from each subject.

^‡^Significant difference between G8 and g2 (*P* < 0.001).

BMI = body mass index; WMDA = well-to-moderately differentiated adenocarcinoma; HGIN = high-grade intraepithelial neoplasia.

### Comparison of Image Quality Between G8 and g2

**FIGURE 3.**
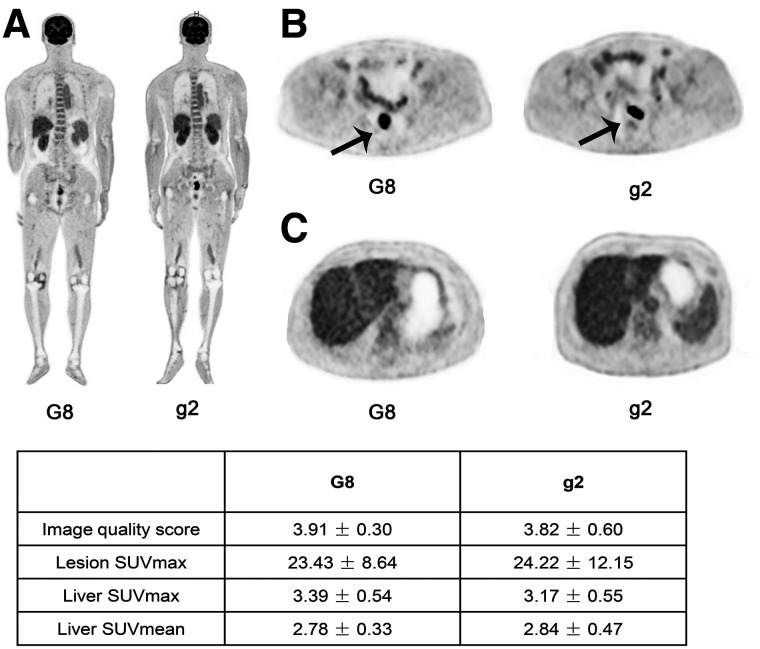
PET images of 63-y-old man with CRC reconstructed in G8 and another 63-y-old man with CRC reconstructed in g2 (A, coronal slice of the whole body; B, transverse view of CRC lesion [arrow]; C, transverse image of liver). Image quality in G8 was comparable to that in g2, which meets standard for clinical diagnosis.

**FIGURE 4.**
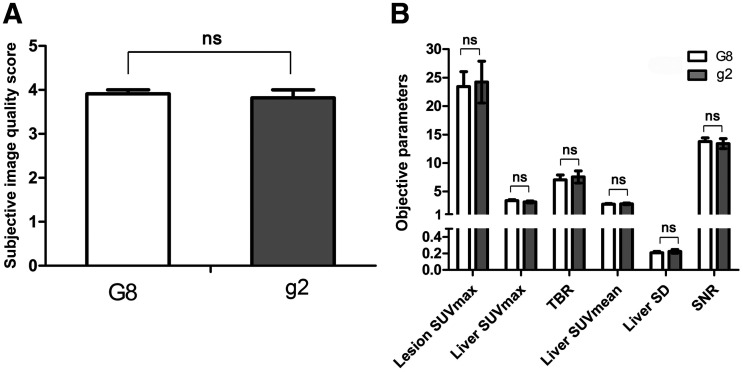
Bar graph of values of subjective image quality score (A) and objective parameters (B) between G8 and g2. Comparable result of qualitative and quantitative analysis was shown between the 2 groups. ns = not significant.

The results from the subjective and objective analyses of image quality in G8 and g2 are shown in Table 6. The visual image quality score in G8 was 3.91 ± 0.30, which was equivalent to that in g2 (3.82 ± 0.60). The lesion SUV_max_ and TBR in G8 (23.43 ± 8.64 and 7.07 ± 2.74) were slightly lower than those in g2 (24.22 ± 12.15 and 7.56 ± 3.51) but without statistical significance (all *P* > 0.05). The liver SUV_mean_ and liver SD were similar in G8 and g2 (liver SUV_mean_ [mean ± SD], 2.78 ± 0.33 vs. 2.84 ± 0.47, and liver SD [noise in liver], 0.21 ± 0.05 vs. 0.23 ± 0.08, respectively). The SNRs in G8 and g2 were 13.77 ± 2.14 and 13.40 ± 2.90, respectively, without a significant difference (*P* = 0.716). None of the quantitative parameters showed significant differences between the groups (all *P* > 0.05), indicating an equivalent performance between the 2 groups (Figs. 3 and 4).

**TABLE 6.** Qualitative Image Quality Score and Quantitative Parameters in the Ultra-Low-Activity Group and Full-Activity Group

Parameter	G8	g2	*P*
Image quality score	3.91 ± 0.30	3.82 ± 0.60	0.311
Lesion SUV_max_	23.43 ± 8.64	24.22 ± 12.15	0.863
Liver SUV_max_	3.39 ± 0.54	3.17 ± 0.55	0.354
Liver SUV_mean_	2.78 ± 0.33	2.84 ± 0.47	0.747
Liver SD	0.21 ± 0.05	0.23 ± 0.08	0.544
TBR	7.07 ± 2.74	7.56 ± 3.51	0.738
SNR	13.77 ± 2.14	13.40 ± 2.90	0.716

Data are presented as mean ± SD. Mean value and SD were calculated on basis of Likert score for each patient. Mean value and SD of other quantitative parameters were calculated on basis of measurement in ROIs.

## DISCUSSION

The 194-cm-long total-body PET/CT system has a spatial resolution of approximately 3.0 mm and a system sensitivity of up to 174 kcps/MBq with NEMA NU-2-2018 (*12*). The system enables excellent image quality and provides new opportunities to assess clinical imaging protocol modifications such as shorter scan durations, low-activity tracer injection, delayed imaging, or repeated scans. The current study assessed the feasibility of using a low-activity ^18^F-FDG injection. The dominant physical characteristic of the total-body PET scanner is its high sensitivity, being 40-fold higher than that of current systems (*13*). The SNR in PET images, representing the image quality, is proportional to the square root of the product of system sensitivity, injected activity, and acquisition time (*14*). For the total-body PET/CT scanner, the data quality is as important as the data quantity (i.e., total true counts). Data quality is often measured as noise-equivalent counts, which are calculated as T^2^/(T + R), where T and R are the trues and randoms, respectively. The random rate is considered to be proportional to the square of the injected activity, whereas the true rate is proportional to the injected activity, so the random rate is approximately 100 times higher in the full-activity situation than in the ultra-low-activity (one tenth of a full-activity dose) situation. As a result, if the ultra-low-activity data and the full-activity data have equivalent total true counts, the ultra-low-activity data would have a higher noise-equivalent count, that is, better data quality. Therefore, it is possible to achieve comparable image quality with a shorter acquisition time than that estimated from the rule of constant product of the acquisition time and the activity. Our previous studies demonstrated the capability of the total-body PET/CT scanner to achieve good image quality with a reduced injected activity of up to one half and one seventh the recommended standard in the clinic (3.7 MBq/kg) (*9**,**15*). However, these studies were on only a small number of cases and did not evaluate both the qualitative and the quantitative aspects. Therefore, the current study aimed to provide qualitative and quantitative assessments of image quality after injection of an ultra-low ^18^F-FDG activity in oncologic patients using the total-body PET/CT scanner.

Our results demonstrated that images taken 8 min after injection of an ultra-low ^18^F-FDG activity provided acceptable image quality for clinical reporting. The liver SUV_mean_ showed good consistency for all PET series, without significant differences between groups among G1–G15. However, the liver SNR showed a lack of significant differences between groups only among G8–G15. Lesion SUV_max_ and TBR in G8 and G10 did not significantly differ from those in G15. On the basis of the above results, an 8-min PET acquisition time with an ultra-low-activity injection protocol could yield diagnostic-level image quality for clinical oncologic applications.

In this study, we found that lesion SUV_max_ increased along with the acquisition time, a finding that was inconsistent with a previous study (*6*). We hypothesized that the additional uptake time (with a maximum of 15 min) will be more noticeable when the acquired counts are reduced. The effect on the increased accumulation of ^18^F-FDG in the malignant lesions will be more significant and thus increase the lesion SUV, as observed in the time–activity curves in previous studies (*16**,**17*). Additionally, a discrepancy between qualitative and quantitative analysis was found. In the quantitative analysis, the TBR and SNR were higher for G10–G15 than for G8, but without statistical differences. The liver SD decreased as the acquisition time increased, but no significant difference was observed between G8 and G15. In this study, we used lesion TBR, liver SNR, and SD as the indices of quantitative image quality. However, the qualitative analysis process was far more complex as it could be influenced by the reader’s experience, preference, and training before the analysis.

In conventional whole-body PET/CT imaging, PET acquisition is performed in a step-and-shoot mode with 6–7 bed positions. The total-body PET/CT imaging uses a 1-step acquisition mode because the 194-cm axial field of view can cover the patient’s entire body in 1 bed position. Our previous study reported that a total-body PET scan with a 2-min acquisition time and an injected activity of 4.4 MBq/kg could yield images superior to the average image quality (*6*). The liver SUV_max_ and SD in the 2 studies showed a similar tendency with the acquisition time, but with different values. This is mainly caused by the difference in the uptake time of the enrolled patients between the 2 studies. Although a 60-s acquisition can maintain the diagnostic performance at a sufficient level, as reported in the previous study (*6*), the injected activity was 18% higher than the full activity in this study. Thus, in the matched-pair part of this study, a 2-min acquisition was selected as the control to evaluate the image quality and feasibility of ultra-low ^18^F-FDG activity in total-body PET/CT imaging. Compared with full activity using 2 min of acquisition, the image quality of ultra-low ^18^F-FDG activity using 8 min of acquisition revealed an equivalent result. The concept of an ultra-low-activity ^18^F-FDG PET scan has several benefits. One is the significant reduction of radiation from the PET radiotracers, which is approximately 7 mSv in a conventional PET whole-body examination (*18**)*. If activity can be reduced to one tenth, it allows for increased use of PET scans in radiation-sensitive populations (infants, children, and adolescents). For pediatric imaging, there are risks associated with the acquisition duration and injected dose. An increased injected activity is associated with an increased risk of radiation-induced cancer in the pediatric population (*19*). According to recently published guidelines, images of diagnostic image quality using the lowest possible dose are desired in pediatric ^18^F-FDG PET/CT for oncology (*5*). The ultra-low ^18^F-FDG activity, with reduced radiation exposure, will provide a more feasible solution for pediatric imaging. In addition, the ultra-low ^18^F-FDG activity is an attractive option for repeated scans for monitoring treatment response. It may become an effective strategy for patient management without concerns about the cumulative absorbed radiation dose.

Our study has several limitations. First, 30 patients with 10 types of cancer were enrolled prospectively in the study. The highest body weight in the enrolled cases was 88 kg. Image quality can be influenced by patient size (weight and body mass index), and image quality might be degraded because of excessive attenuation in heavier patients (*5*). Additionally, only patients with CRC were validated in the matched study. Although they were well matched on the basis of the demographic and pathologic features, some marginal differences remained. The relatively small number of patients enrolled in the matched study meant that there was a potential selection bias. Second, although ^18^F-FDG is the most widely used radiotracer in oncologic studies, it is not applicable to all types of cancer, because not all tumors are ^18^F-FDG–avid. Furthermore, the extent of ^18^F-FDG uptake is easily affected by certain factors. Respiratory motion might blur the lesions on which the impact of the SUV measurement may differ with different acquisition times (*20*). We selected lesions at least 10 mm in diameter (measured on CT images), for which the error induced by the respiratory motion could be minimized. Finally, the 2-dimensional ROI did not necessarily capture the true SUV_max_ of the whole tumor volume, as limited by the current measurement software. The reconstruction parameters used in this study were the same as those with the standard activity used in our department without specific modification. However, these parameters were based on the high counts and the clinical requirements for diagnosis. To improve lesion detection, we applied point-spread-function modeling in the PET reconstruction, the same as in routine practice, which may cause a bias in the quantitative estimate. Future studies should compare the point-spread-function reconstruction with the non–point-spread-function reconstruction, as well as investigate the optimal reconstruction parameters for the ultra-low ^18^F-FDG activity.

## CONCLUSION

The study demonstrated that use of an ultra-low ^18^F-FDG activity (0.37 MBq/kg) in total-body PET/CT was feasible for oncologic studies, with a clinical diagnostic-level image quality achieved. Further investigation will be performed to explore the optimal reconstruction parameters for an ultra-low ^18^F-FDG activity in the clinic.

## Notes

KEY POINTS **QUESTION:** Does an ultra-low ^18^F-FDG activity (0.37 MBq/kg) in total-body PET/CT compromise image quality? **PERTINENT FINDINGS:** This study demonstrated that the image quality seen using an ultra-low ^18^F-FDG activity in total-body PET/CT meets clinical requirements. **IMPLICATIONS FOR PATIENT CARE:** Total-body PET/CT imaging with an ultra-low ^18^F-FDG activity can be used in radiation-sensitive populations such as infants, children, and adolescents, as well as in patients who need repeated scans to monitor treatment response.
